# Receptor Binding Domains of TcdB from *Clostridioides difficile* for Chondroitin Sulfate Proteoglycan-4 and Frizzled Proteins Are Functionally Independent and Additive

**DOI:** 10.3390/toxins12120736

**Published:** 2020-11-24

**Authors:** Daniel Henkel, Helma Tatge, Dennis Schöttelndreier, Liang Tao, Min Dong, Ralf Gerhard

**Affiliations:** 1 Institute of Toxicology, Hannover Medical School, 30625 Hannover, Germany; henkel.daniel@mh-hannover.de (D.H.); tatge.helma@mh-hannover.de (H.T.); schoettelndreier.dennis@mh-hannover.de (D.S.); 2 Department of Urology, Boston Children’s Hospital, Boston, MA 02115, USA; taoliang@westlake.edu.cn (L.T.); min.Dong@childrens.harvard.edu (M.D.); 3 Departments of Surgery and Microbiology, Harvard Medical School, Boston, MA 02115, USA; 4 Institute of Basic Medical Sciences, Westlake Institute for Advanced Study, Westlake University Hangzhou, Hangzhou 310000, China

**Keywords:** *Clostridioides difficile*, glucosyltransferase, receptor binding

## Abstract

Toxin B (TcdB) produced by *Clostridioides difficile* is a main pathogenicity factor that affects a variety of different cell types within the colonic mucosa. TcdB is known to utilize frizzled-1,2,7 and chondroitin sulfate proteoglycan-4 (CSPG4) as protein receptors. By using human cervical cancer cell line HeLa CSPG4 knockout (CSPG4^−/−^) cells as well as TcdB mutants which do not bind to either CSPG4 or frizzled-1,2,7, or both, we evaluated the impact of the individual receptors for cytopathic and cytotoxic effects of TcdB. We compared TcdB from the reference strain VPI10463 (TcdB_VPI_) and the endemic strain R20291 (TcdB_R20_) which does not interact with frizzled-1,2,7. TcdB_VPI_ devoid of CSPG4 binding (TcdB_VPI_ ΔCROP) shows identical cytopathic potency as full-length TcdB in HeLa CSPG4^−/−^ cells, indicating that interaction with frizzled proteins is not affected in the presence of the C-terminal CROP domain. We validated CSPG4 as cellular receptor for both TcdB toxinotypes in HeLa and HEp-2 cells. By exchange of a single phenylalanine residue, 1597 with serine, we generated a mutated TcdB_VPI_ variant (TcdB_VPI_ F1597S) that in accordance with TcdB_R20_ lacks binding to frizzled-1,2,7 and showed identical potency as TcdB_R20_ on HeLa cells. This enabled us to estimate the respective share of CSPG4 and frizzled-1,2,7 in the cytotoxic and cytopathic effect induced by TcdB. Our data reveal that binding to frizzled-1,2,7 and to CSPG4 occurs independently and in an additive manner.

## 1. Introduction

Infections with pathogenic *Clostridioides difficile*, a gram-positive and spore-forming bacterium, may lead to severe diarrhea and pseudomembranous colitis [1]. Antibiotic treatment changes the commensal intestinal flora, leading to germination and proliferation of *C. difficile. C. difficile* produces two homologous exotoxins, Toxin A (TcdA) (308 kDa) and Toxin B (TcdB) (270 kDa), that are major virulence factors responsible for diseases associated with *C. difficile* infection. Both TcdA and TcdB are large glucosyltransferases that inactivate small GTPases of the Rho family within the target cell, leading to the disruption of the actin cytoskeleton and, thus, to inhibition of actin-dependent processes. To accomplish this intracellular task, TcdA and TcdB, as well as all other homologous large clostridial glucosyltransferases, comprise different functional domains that are summarized in the ABCD structure type: (A) The N-terminal enzymatically active glucosyltransferase domain (GTD), (B) the C-terminal combined repetitive oligopeptides (CROP), which may mediate binding to cells (designated receptor-binding domain 1, RBD1), (C) the protease domain for the autoproteolytic release of the GTD and (D) the delivery domain that mediates translocation of the GTD across the endosome membrane. Experiments with C-terminally truncated toxins (TcdA ∆CROP and TcdB ∆CROP) indicate that the delivery domain contains a second receptor-binding domain (RBD2) [2,3]. Some receptors or binding structures have already been identified that contribute to uptake of large clostridial glucosyltransferases: For TcdA the carbohydrate structures Gal-α-(1,3)-Gal-β-(1,4)-GlcNAc [4] as well as sulfated glycosaminoglycans and the low-density lipoprotein receptor (LDLR) [5] were described. For Large enterotoxin from *C. perfringens* (TpeL) the low-density lipoprotein receptor-related protein 1 (LRP1) was identified [2]. Very recently, semaphorins 6A and B were identified as receptors for the Lethal Toxin from *P. sordellii* (TcsL) [6,7]. Three proteins were identified to serve as receptors for TcdB: Chondroitin sulfate proteoglycan-4 (CSPG4/NG-2) [8,9], Wnt-receptors frizzled-1,2,7 (FZD1,2,7) [9] and poliovirus receptor-like 3 (PVRL3/Nectin 3) [10]. FZD1,2,7 were shown to be the major receptors in the colonic epithelium, which are bound by the RBD2 (aa 1285-1804) [9,11]. CSPG4-binding region remains to be defined, although the presence of the N-terminal region of CROP (approximately aa 1810–1850) is required [8,9,11,12]. Whether PVRL3 acts as a functional receptor for TcdB still has to be established. Although direct interaction of TcdB (aa 1372–1493) with the extracellular domain of PVRL3 was shown [13,14], PVRL3 knockdown did not affect potency of TcdB in terms of cell rounding in the human cervical cancer cell line HeLa but only reduced toxin-induced necrosis [10]. A lot of knowledge about functional domains of TcdB and other large glucosyltransferases has been accumulated within the past years, and the structure of full-length TcdB has recently been resolved [15]. Yet, it is still unclear whether there is a hierarchy in receptor binding or if binding to frizzled proteins and CSPG4 occurs synergistically or independently from each other. Furthermore, it has been reported that TcdB from hypervirulent endemic *C. difficile* strain (NAP1/027, clade 2) does not bind to frizzled proteins due to specific mutations within the RBD2 [14,16]. Therefore, the aim of our work was to examine both the dynamic and the dependency of the two established functional receptors described for uptake of TcdB.

## 2. Results

### 2.1. HeLa CSPG4^−/−^ Cells Are Less Susceptible to Both TcdB_VPI_ and TcdB_R20_

**Figure 1:**
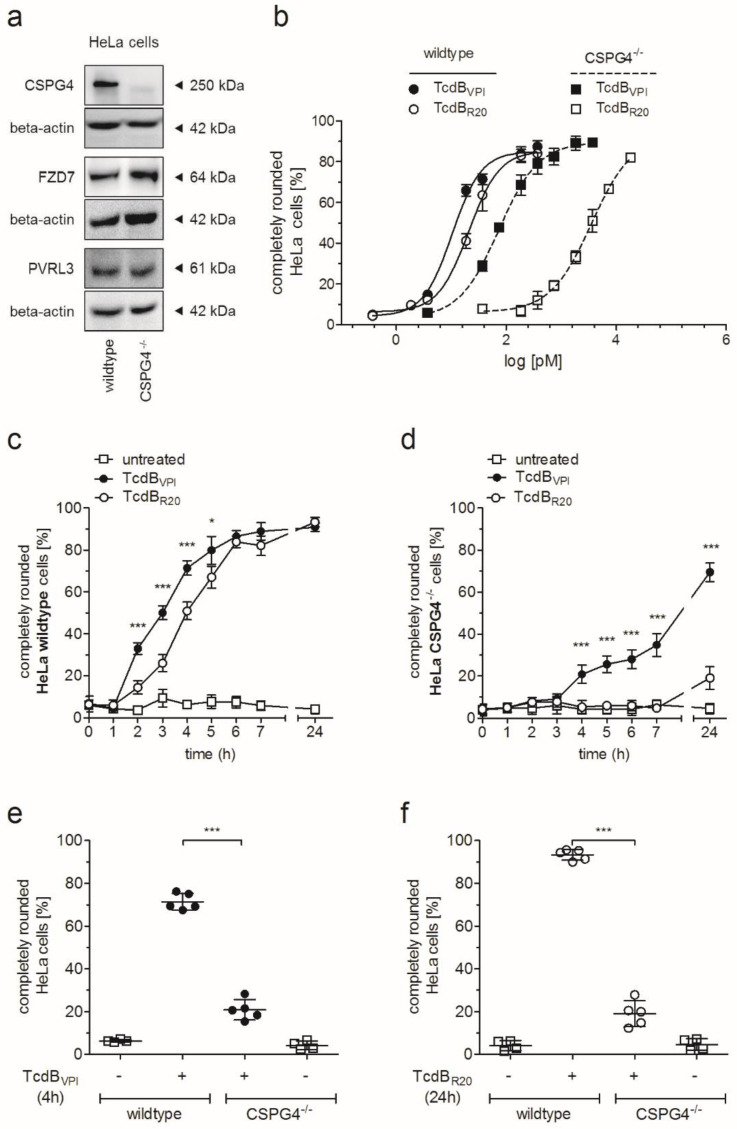
Chondroitin sulfate proteoglycan-4 (CSPG4) is the predominant receptor for Toxin B (TcdB) in HeLa cells. (**a**) Immunoblot analysis of representative TcdB-interacting proteins CSPG4, frizzled-7 (FZD7), and poliovirus receptor-like 3 (PVRL3) in HeLa wildtype and CSPG4^−/−^ whole cell lysates. (**b**) Concentration-dependent analysis of potency of TcdB_VPI_ and TcdB_R20_ towards HeLa cells. EC50 of TcdB_VPI_ was 10 pM and 73 pM and of TcdB_R20_ 21 pM and 3517 pM in HeLa wildtype and CSPG4^−/−^, respectively (means ± SD, *n* = 4). (**c**) Time-dependent cell rounding of HeLa wildtype (left graph) and CSPG4^−/−^ cells (right graph) treated with 10 pM TcdB_VPI_ or TcdB_R20_ (means ± SD, *n* = 5). (**d**) Cell rounding induced by 10 pM TcdB_VPI_ is significantly reduced from 70% in wildtype cells to 20% in CSPG4^−/−^ cells after four hours treatment. Cell rounding induced by 10 pM TcdB_R20_ is also significantly reduced in CSPG4^−/−^ cells. Residual cell rounding effect in CSPG4^−/−^ cells was observed as early as 24 h after addition of toxin. (**e**,**f**) Certain time-points from (**b**) and (**c**) are shown to highlight the impact of CSPG4. Shown are means ± SD, *n* = 5, * <0.05; *** <0.001.

To evaluate the role of CSPG4 on toxin uptake into cells we performed time- and concentration-dependent cell rounding assay. Figure 1a shows immunoblot analysis of the indicated proteins in HeLa wildtype and CSPG4^−/−^ cells. Knockout of CSPG4 did not affect abundance of frizzled-7 (FZD7) and PVRL3 relative to beta-actin content. Treatment of HeLa cells revealed that both, reference TcdB (TcdB_VPI_, historic strain VPI10463) as well as TcdB from strain R20291 (TcdB_R20_) showed greatly reduced activity on CSPG4^−/−^ cells compared to HeLa wildtype cells (Figure 1b). Whereas the EC50 of TcdB_VPI_ and TcdB_R20_ in HeLa wildtype cells were 10.4 pM and 20.7 pM, respectively, the EC50 values increased to 72.9 pM and 3517 pM for TcdB_VPI_ and TcdB_R20_. Obviously, CSPG4 is a prominent receptor for both TcdB reducing potency of TcdB_VPI_ about 7-fold and of TcdB_R20_ about 170-fold when absent in HeLa cells. The derived EC50 values indicate a higher relevance of CSPG4 in uptake of TcdB_R20_ compared to TcdB_VPI_.

The impact of CSPG4 for TcdB_VPI_ and TcdB_R20_ is highlighted in Figure 1c,d where the time course of cell rounding induced by 10 pM toxin in HeLa wildtype and CSPG4^−/−^ cells are shown. Cell rounding of 50% HeLa wildtype cells induced by TcdB_R20_ was delayed for about one hour compared to TcdB_VPI_. In HeLa CSPG4^−/−^ cells, cell rounding effect of TcdB_R20_ was delayed for even 20 h. Selected time points for TcdB_VPI_ (4 h) and TcdB_R20_ (24 h) were chosen to illustrate the delay to induce equipotent cell rounding by both toxins (Figure 1e,f). These data substantiate CSPG4 as primary receptor for uptake of TcdB in HeLa cells, but also reveal significant differences in uptake of TcdB_VPI_ and TcdB_R20_ to receptors others than CSPG4.

### 2.2. RBD2 Is Not Affected by CSPG4 Interaction or the CROP Domain

**Figure 2:**
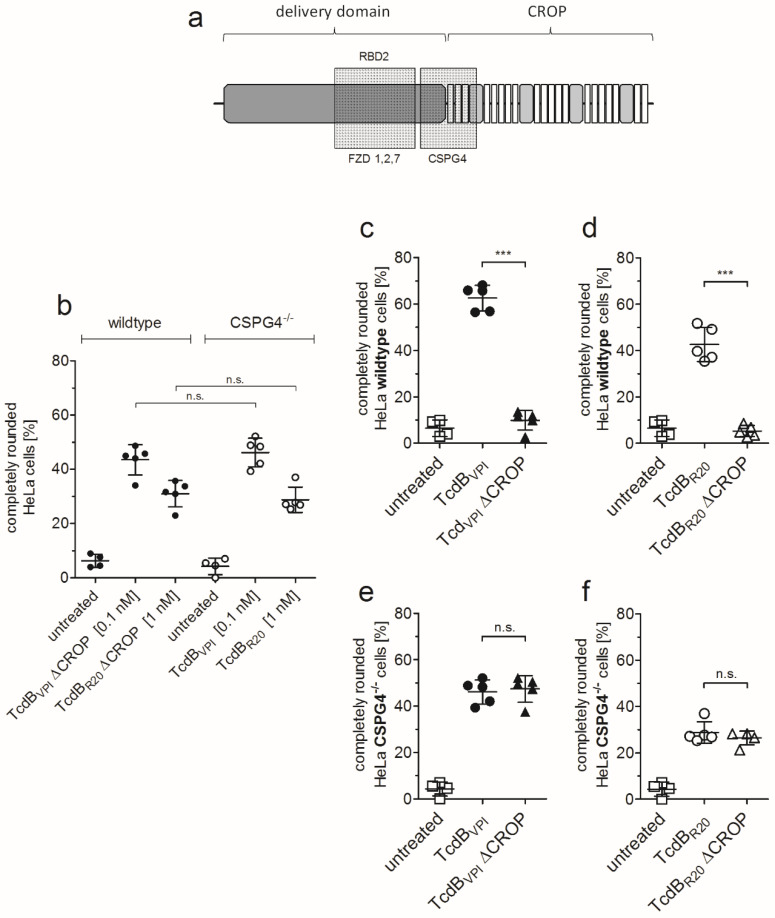
The combined repetitive oligopeptide (CROP) domain does not interfere with receptor-binding domain (RBD2). (**a**) Scheme of the C-terminal part of TcdB. The delivery domain (amino acids 900–1833) and the CROP domain (aa 1834-2366) are independent structural domains. Whereas the frizzled binding domain (aa 1285–1804) is a subdomain of the delivery domain. The hinge region (aa 1792-1834) downstream of the FZD binding domain contributes to CSPG4 binding. (**b**) Deletion of the CROP domain functionally abolishes CSPG4-binding. The effect of TcdB_VPI_ on CSPG4^−/−^ cells is exactly the same as TcdB_VPI_ ΔCROP (each 0.1 nM), lacking a functional CSPG4-binding domain. The same is observed for TcdB_R20_ and TcdB_R20_ ΔCROP (each 1 nM). (**c**,**d**) Cell rounding assay using 10 pM full-length TcdB_VPI_ or TcdB_R20_ as well as 10 pM TcdB_VPI_ ΔCROP or TcdB_R20_ ΔCROP applied to HeLa wildtype cells. (**e**,**f**) 100 pM full-length TcdB_VPI_ and TcdB_VPI_ ΔCROP as well as 1 nM TcdB_R20_ and TcdB_R20_ ΔCROP show the same potency on HeLa CSPG4^−/−^ cells. The toxin concentrations were chosen to allow detection of both increase and decrease in number of round cells after four hours. Shown are means ± SD, *n* = 5 in all experiments, *** <0.001, n.s. non-significant differences.

To further evaluate CSPG4 binding in a complementary approach, we deleted the amino acids 1831–2366 in TcdB, known as CROP domain (TcdB ΔCROP) to functionally inactivate CSPG4 binding of TcdB [9]. TcdB ΔCROP still harbors a functional FZD binding domain (RBD2) which is entirely located within the delivery domain (Figure 2a).

In this way we examined whether binding to frizzled proteins via RBD2 is affected in the presence or absence of the CROP domain and, in consequence to CSPG4 binding. Figure 2b shows that TcdB_VPI_ ΔCROP shows the same potency in HeLa wildtype cells as full-length TcdB in CSPG4^−/−^ cells when applied at identical concentration (0.1 nM). This is also true for TcdB_R20_ and TcdB_R20_ ΔCROP, although these toxins have to be applied at 10-fold higher concentration (1 nM) to achieve a similar effect compared to TcdB_VPI_. Deletion of functional CSPG4 binding strongly reduced cytopathic effect of TcdB_VPI_ (Figure 2c). This is also true for TcdB_R20_, which has not been reported before (Figure 2d). When TcdB_VPI_ ΔCROP was applied to CSPG4^−/−^ cells it showed identical effect in cell rounding as full-length toxin. Obviously, the absence of the CROP domain or lack of CSPG4 affected potency of TcdB to the same amount (Figure 2e). This principle is also true for TcdB_R20_ (Figure 2f). These data again confirm CSPG4 as main receptor for TcdB_VPI_ as well as for TcdB_R20_ on HeLa cells but also proves that binding to CSPG4 and frizzled proteins occur via independent binding regions that do not interfere with each other. Moreover, in absence of CSPG4, the presence or absence of the CROP domain does not affect uptake of TcdB_VPI_ via RBD2. Binding of CSPG4 is therefore neither required for unmasking of RBD2 nor does it facilitate toxin uptake via FZD-binding domain.

### 2.3. RBD2 of TcdB_VPI_ But Not of TcdB_R20_ Binds to FZD1,2,7

**Figure 3:**
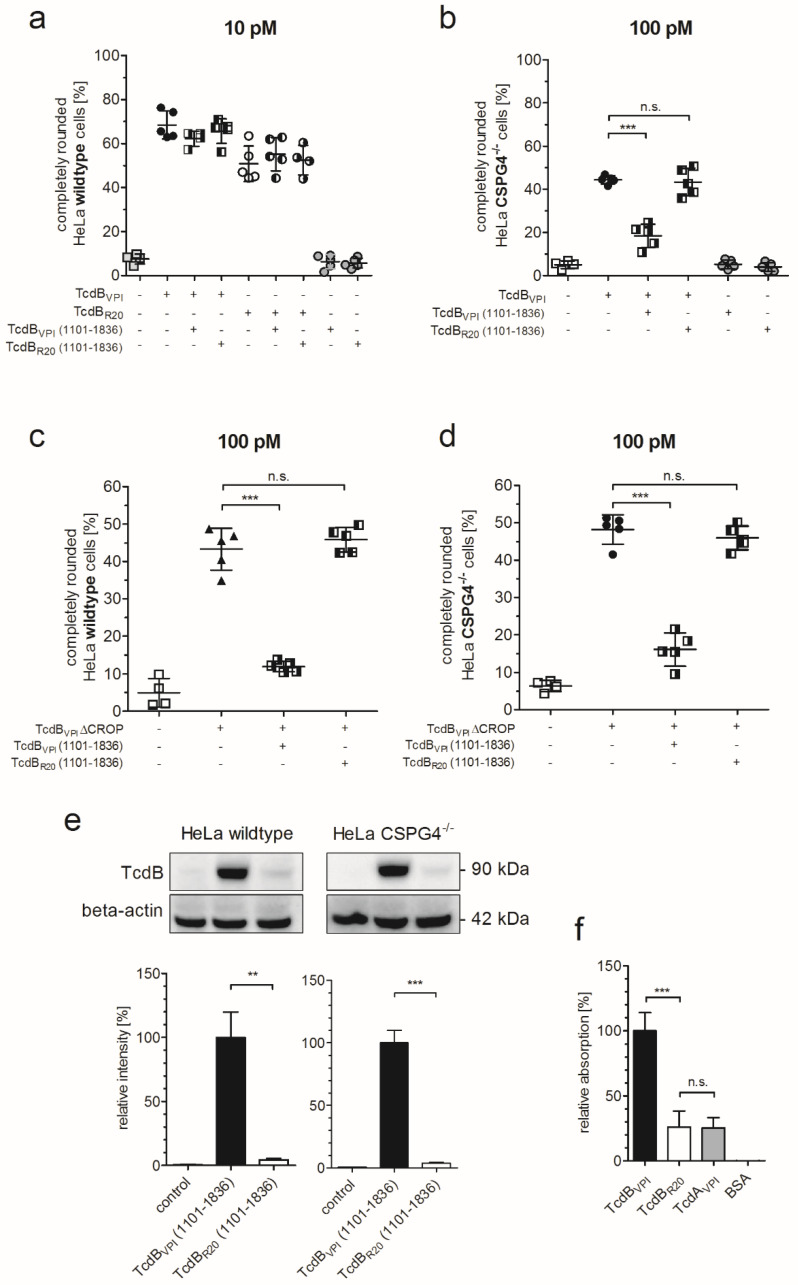
RBD2 of TcdB_VPI_, but not of TcdB_R20_ binds to HeLa cells via FZD1,2,7. (**a**) Cell rounding of HeLa wildtype cells induced by 10 pM TcdB_VPI_ or TcdB_R20_ after preincubation of cells with TcdB fragment (aa 1101–1836, comprising FZD-binding domain) of TcdB_VPI_ or TcdB_R20_ in 1000-fold excess (10 nM). (**b**–**d**) Cell rounding of indicated cells induced by indicated TcdB after preincubation of cells with indicated TcdB fragment in 1000-fold excess. (**e**) Immunoblot and evaluation of binding of 1 µg TcdB_VPI_ (1101–1836) as well as TcdB_R20_ (1101–1836) representing the FZD-binding domain (RBD2) to HeLa wildtype (left panel) and CSPG4^−/−^ cells (right panel) (*n* = 3). (**f**) Evaluation of binding of FZD2~Fc to immobilized TcdB_VPI_, TcdB_R20_ and TcdA_VPI_ in ELISA (*n* = 6), ** <0.01, *** <0.001, n.s. non-significant differences.

The significant difference in cytopathic effect of TcdB_VPI_ and TcdB_R20_ towards HeLa CSPG4^−/−^ cells implicates that the RBD2 of both toxins do not bind to the same receptor which also was hypothesized by López-Ureña and co-workers [16]. To directly examine this hypothesis, we tested whether the extended RBD2 of both toxins comprising the isolated TcdB fragment aa1101–1836 competes with full-length and CROP-depleted toxins in HeLa wildtype and CSPG4^−/−^ cells. The extended RBD2 of TcdB_VPI_ slightly, but non-significantly, reduced the number of round wildtype cells when applied in 1000-fold excess in combination with TcdB_VPI_. The extended RBD2 of TcdB_R20_ had no effect at all in competing with TcdB_VPI_ (Figure 3a). The competition was highly significant when TcdB_VPI_ together with 1000-fold excess of its respective extended RBD2 was applied to HeLa CSPG4^−/−^ cells (Figure 3b). The same is true for corresponding experiments using TcdB_VPI_ ΔCROP on HeLa wildtype or CSPG4^−/−^ cells (Figure 3c,d). In contrast, the extended RBD2 of TcdB_R20_ did not compete with TcdB_VPI_ on CSPG4^−/−^ cells, nor did it reduce TcdB_VPI_ ΔCROP-induced cell rounding on HeLa wildtype or CSPG4^−/−^ cells. The findings that the extended RBD2 of TcdB_R20_ did not affect uptake of TcdB_VPI_ implicates that it does not bind to FZD1,2,7. We therefore tested binding of the RBD2 of TcdB_R20_ to HeLa cells in immunoblots. In contrast to TcdB_VPI_ the extended RBD2 of TcdB_R20_ does neither bind to HeLa wildtype nor to HeLa CSPG4^−/−^ cells (Figure 3e). We previously observed reduced recognition of TcdB from endemic strain R20291 by a monoclonal antibody raised against the region 1128–1852 of TcdB_VPI_ [14]. To avoid a bias due to differences in recognition of toxins from different clades, we tested our affinity purified polyclonal anti-TcdB IgG accordingly. (Figure S1). Although recognition of TcdB_R20_ (1101–1836) by anti-TcdB is about three-fold less than recognition of TcdB_VPI_ (1101–1836), our results nevertheless show lack of prominent binding of TcdB_R20_ to HeLa cells. Binding of frizzled protein to different toxins was additionally checked in a complementary enzyme linked immunosorbent assay (ELISA) to avoid anti-TcdB based bias: the extracellular domain of frizzled-2 bound to immobilized TcdB_VPI_ but not to TcdB_R20_ or the negative control TcdA_VPI_ (Figure 3f).

### 2.4. Exchange of Phenylalanine 1597 in TcdB_VPI_ Abolishes Frizzled Binding

**Figure 4:**
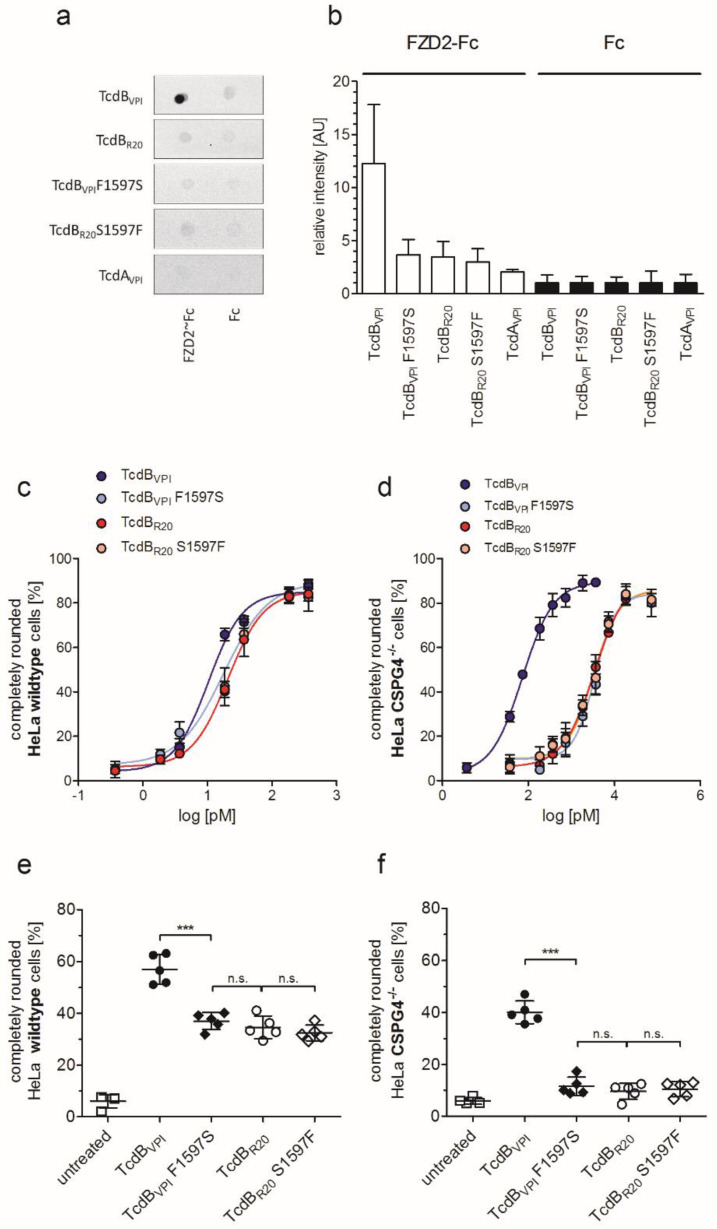
Point mutation F1597S reduces the potency of TcdB_VPI_ to the level of TcdB_R20_. (**a**) Representative dot blots from three separate experiments show binding of indicated toxins to immobilized FZD2~Fc and to Fc as controls. (**b**) Statistical analysis of all dot blots as shown in (**a**) (*n* = 3). (**c**,**d**) Concentration-dependent analysis of potency of mutated TcdB_VPI_ and TcdB_R20_ towards HeLa cells. Mutation of F1597S decreases the effect of TcdB_VPI_ to the level of TcdB_R20_ on HeLa wildtype and CSPG4^−/−^ cells. Mutation of S1597F of TcdB_R20_ did not reconstitute the potency to the level of TcdB_VPI_ on both HeLa wildtype and CSPG4^−/−^ cells. EC50 of TcdB_VPI_ F1597S was 18 pM and 3551 pM and of TcdB_R20_ S1597F 21 pM and 3290 pM in HeLa wildtype and CSPG4^−/−^, respectively (means ± SD, *n* = 4). (**e**,**f**) Cell rounding assays for comparison of TcdB_VPI_ and TcdB_R20_ and their reciprocal mutations TcdB_VPI_ F1597S and TcdB_R20_ S1597F. Inhibition of frizzled-1,2,7 binding by exchange of F1597 reduced potency of TcdB_VPI_ to the level of TcdB_R20_. Toxins were applied at 10 pM to HeLa wildtype cells and 100 pM to CSPG4^−/−^ cells for four hours, *** <0.001, n.s. non-significant differences.

From the co-crystal structure of TcdB in complex with the extracellular domain of FZD2 it was clear that F1597 is essential for binding of TcdB to FZD1,2,7 [11]. This key residue is changed to serine in TcdB_R20_. To examine whether loss of FZD-binding in TcdB_R20_ could be due to sequence variations within the FZD-binding domain, we generated the F1597S mutant of TcdB_VPI_ and the complementary mutant S1597F of TcdB_R20_. Dot blot analyses revealed that FZD2 only bound to TcdB_VPI_, but to not to TcdB_VPI_ F1597S or TcdB_R20_ (Figure 4a,b). A single exchange of S1597 for phenylalanine however did not reconstitute binding of TcdB_R20_ to FZD2, indicating that additional divergence in amino acid sequence between TcdB_VPI_ and TcdB_R20_ contributes to receptor specificity. Equal sensitivity and specificity of purified polyclonal anti-TcdB IgG towards different toxinotypes is shown in Supplementary Materials (Figure S1). As can be assumed, exchange of F1597 reduced the cytopathic effect of 10 pM TcdB_VPI_ (TcdB_VPI_ F1597S) towards wildtype cells exactly to the level of TcdB_R20_, while mutation of S1597F in TcdB_R20_ did not increase the potency of TcdB_R20_ (Figure 4c,e). The impact of frizzled binding is even more prominent in CSPG4^−/−^ cells. There, a 10-fold higher concentration was necessary to induce 40% cell rounding by TcdB_VPI_ after four hours compared to wildtype cells. TcdB_VPI_ F1597S, TcdB_R20_ and TcdB_R20_ S1597F all were significantly less potent, inducing cell rounding of about 10% of cells (Figure 4d,f).

### 2.5. CSPG4 Is the Primary Receptor Facilitating Cytotoxic Effect of TcdB in HeLa cells

**Figure 5:**
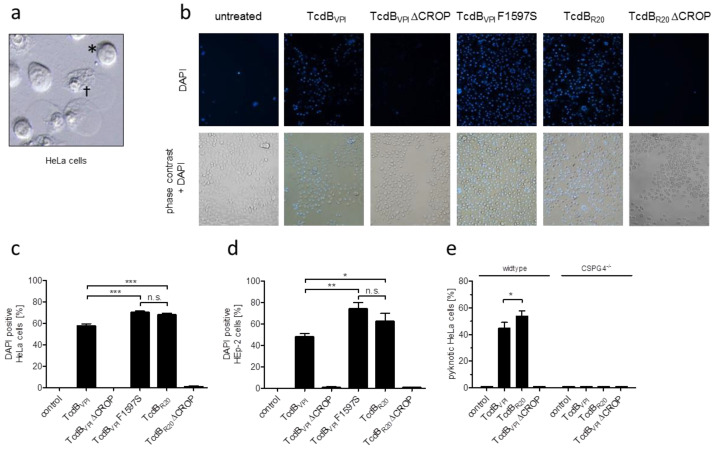
CSPG4 mediates the cytotoxic effect of both TcdB_VPI_ and TcdB_R20_ on HeLa and HEp-2 cells. (**a**) Representative micrograph of rounded (*) and pyknotic (†) HeLa wildtype cells after treatment with 10 nM TcdB. Pyknotic cells show condensed chromatin and huge blister. (**b**) DAPI incorporation marks pyknotic cells as non-viable. Overlay of phase contrast and fluorescence micrographs show ratio of rounded and pyknotic cells after treatment with 10 nM of indicated toxins. (**c**) Statistical analysis of DAPI-positive HeLa cells after treatment with 10 nM of indicated toxins. (**d**) Statistical analysis of DAPI-positive HEp-2 cells after treatment with 4 nM of indicated toxins. (**e**) Early cell death (pyknosis) induced by 10 nM of indicated toxins in HeLa wildtype and CSPG4^−/−^ cells. Shown are means ± standard deviations, *n* = 4 in all experiments, * <0.05; ** <0.01; *** <0.001, n.s. non-significant differences.

At high concentrations TcdB induces early, necrotic cell death which is characterized by chromatin condensation and blistering and therefore termed pyknosis (Figure 5a) [17]. By applying our TcdB variants that selectively interact with frizzled proteins, CSPG4 or both receptor types, we reevaluated the early cell death induced by TcdB to identify the receptor that is mainly involved in facilitating this cytotoxic effect on HeLa cells. TcdB_VPI_ (binds FZD1,2,7 and CSPG4), TcdB_VPI_ ΔCROP (binds FZD1,2,7), TcdB_VPI_ F1597S (binds CSPG4), TcdB_R20_ (binds CSPG4), and TcdB_R20_ ΔCROP (does neither bind to FZD1,2,7 nor to CSPG4) were applied to HeLa wildtype and CSPG4^−/−^ cells. Figure 5b shows 4′,6-diamidino-2-phenylindole (DAPI) incorporation as read out for pyknotic cells, as reported earlier [18]. Only TcdB_VPI_ ΔCROP and TcdB_R20_ ΔCROP, both lacking CSPG4 binding, failed to induce early cell death in this model cell line, albeit complete cell rounding was observed in all samples by phase contrast microscopy (Figure 5b). TcdB_VPI_, TcdB_VPI_ F1597S and TcdB_R20_, which all bind CSPG4, showed cytotoxic effect, whereby both toxin variants that do not bind FZD1,2,7 (TcdB_VPI_ F1597S and TcdB_R20_) even were significantly more cytotoxic (Figure 5c). The same was true for HEp-2 cells, a cell line we used in former studies to characterize pyknotic cell death (Figure 5d) [17,18]. This was cross checked by applying the set of toxins to HeLa CSPG4^−/−^ cells. There, in contrast to wildtype cells, none of the toxins induced early cell death when applied at 10 nM (Figure 5e).

## 3. Discussion

**Figure 6:**
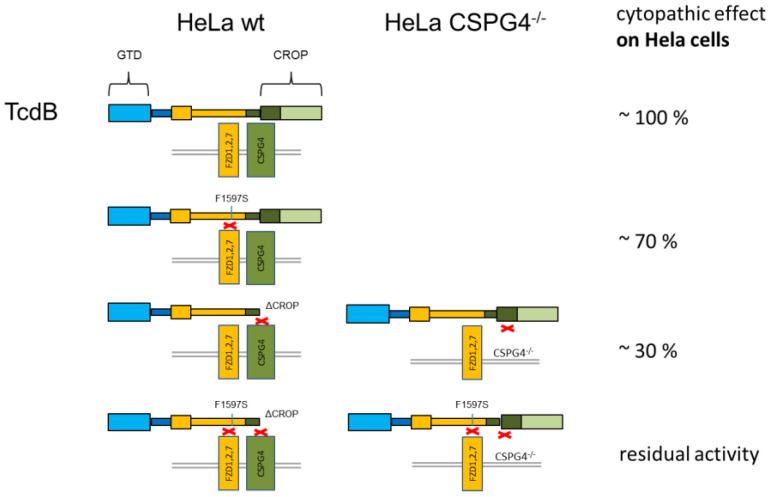
Scheme of TcdB_VPI_ and TcdB_R20_ variants with their estimated share in cytopathic effect on HeLa cells. This scheme summarizes the findings obtained by use of toxin mutants specific for different receptors and by use of HeLa CSPG4^−/−^ cells.

This study evaluates CSPG4 and FZD1,2,7 as receptors for uptake of TcdB into target cells. Our findings show that residual uptake of TcdB is negligible in the absence of CSPG4 and without a functional FZD-binding domain. PVRL3 was described as further receptor being in charge of mediating the cytotoxic effect of TcdB [10]. PVRL3 does not appear to play a significant role in endocytosis of TcdB in our experimental set up using HeLa and HEp-2 cells. Based on this conclusion, we focused on CSPG4 and FZD1,2,7 to estimate the role of both receptor types for the cytopathic and cytotoxic effect of TcdB. We performed complementary experiments with receptor knockdown in a target cell on one hand and functional inactivation of specific receptor binding domains in TcdB on the other hand. We estimated CSPG4 being responsible for mediating roughly 70% toxin uptake into HeLa cells and FZD1,2,7 for about 30%. Moreover, both receptor binding domains act independently, meaning an additive but not synergistic mode of action (Figure 6). We chose whole cell assays for our study since uptake of TcdB is a complex, yet not fully understood, process. Although simultaneous binding of FZD and CSPG4 to TcdB was shown in a bio-layer interferometry (BLI) assay [9], it is not clear whether binding of TcdB to the cell surface happens the same way. For instance, we also observed binding of the glucosyltransferase domain (GTD) of TcdB to the cell surface [19], which might directly or indirectly also contribute to cell adhesion or receptor recognition. For this reason, we omitted binding assays for different toxins but focused on cell rounding as an acknowledged surrogate marker for a rather complex uptake process. This also confirms the fact that mere binding to a receptor does not necessarily mean uptake as long as the receptor is not endocytosed [20].

It has to be emphasized that CSPG4 is overexpressed in transformed cells, meaning that the situation in vivo is supposedly different. CSPG4 was first identified as a high molecular weight melanoma-associated antigen (HMW-MMA) and its rat orthologue termed nerve/glial antigen 2 (NG2) [21,22]. This cell membrane protein is in focus as a target for immunotherapy since it is involved in cell adhesion and migration [23,24,25]. CSPG4 is not expressed in intestinal epithelial cells. We previously showed that TcdB affects Caco-2 cells preferentially from the basolateral side [14]. Since the extracellular domain of FZD2 reduced TcdB effects from basolateral but not from apical in that former study, FZD-proteins are assumed to be located at the basal site of the epithelium. The molecular interaction of FZD2 and TcdB is known in detail [9,11,26], and especially phenylalanine 1597 was identified to be essential for their interaction [11]. Interestingly, TcdB_R20_ shows a serine at position 1597, abolishing binding to FZD1,2,7 [14,16]. Our present findings reveal CSPG4 as predominant receptor for TcdB_VPI_ as well as for TcdB_R20_, and FZD1,2,7 as receptor for only TcdB_VPI_.

Focusing on one receptor might not only define cell specificity, but also alters quality of TcdB-induced effects. By applying TcdB mutants that selectively bind CSPG4 we found significantly higher rate of pyknotic cells, which is a specific cytotoxic effect induced by TcdB that is non-apoptotic and independent of mono-glucosylation of Rho GTPases [17,27] and occurs within few hours. We previously showed that TcdB lacking the CROP and therefore a functional CSPG4 binding site does not provoke early cell death in HEp-2 cells [17]. Our hypothesis is that the fast and efficient uptake of TcdB is a precondition for triggering early cell death. A certain amount of toxin has to be internalized before a significant amount of Rac1 is inhibited by toxin-catalyzed glucosylation to prevent pyknotic effect [18]. If FZD1,2,7 binding of TcdB_VPI_ is prohibited, the cytotoxic effect is increased to the level of TcdB_R20_. One possibility is that uptake of toxin via CSPG4 is more efficient than via FZD in HeLa cells, meaning that binding to FZD sequesters TcdB in a way that overall uptake is slowed down, as long as not all receptors are occupied. Cell rounding might reduce the cytotoxic effect by decreasing cell surface, and thus, by slowing down further intracellular toxin supply, which could be the case for HeLa cells, as well as for the similar cell line HEp-2 [18]. We hypothesize that any receptor that mediates sufficient rapid uptake of TcdB contributes to cytotoxic effect. In fact, in mouse hippocampal neuronal HT-22 cells we observed that TcdB_VPI10463_ F1597S showed reduced cytopathic effect, implicating that FZD-dependent uptake of TcdB also contributes to cytotoxic effect under specific conditions (data not shown). In summary, cytotoxic effect of TcdB is initiated by rapid uptake via CSPG4 (HeLa, HEp-2 cells), whereas the role of PVRL3 seems to be different. The intracellular domain of PVRL3 binds to Afadin. This complex does interact with the platelet derived growth factor (PDGF)-receptor resulting in activation of the PI3K/Akt signaling pathway and thus in preventing apoptosis [28]. As a consequence, knock-down of PVRL3 might promote apoptosis with decreased ATP content in cells therefore resulting in relatively reduced ATP release as measured by Chumbler and co-workers as endpoint for necrosis [10].

We here evaluated CSPG4 as well as FZD1,2,7 as the predominant receptors for TcdB variants. In our HeLa cell system TcdB showed residual activity in terms of cell rounding even in the absence of CSPG4 and FZD binding. The residual activity might be due to uptake via micropinocytosis or by an unknown further receptor or possibly by PVRL3. Particularly, the uptake of TcdB_R20_ is of interest, since the point mutations found in the FZD-binding domain are conserved within all clade 2 TcdB and not randomly distributed to lose interaction with FZD1,2,7 [29]. Mileto and coworkers recently showed that colonic stem cells are equally affected by reference and hypervirulent TcdB expressing *C. difficile* strains, although TcdB_R20_ was applied at much higher concentrations [30]. It remains to be shown whether colonic stem cells express an additional receptor for TcdB_R20_ or if these cells simply overexpress CSPG4 thereby levelling sensitivity towards both TcdB variants. It further remains to be shown whether this model of two independent and additive receptor binding is also true for TcdA. Several binding structures and receptors have been shown to contribute to uptake of TcdA in a crop-dependent and independent manner [3,5,31]. Yet, the exact binding region was only hypothesized for the carbohydrate moiety, but not for other interaction partners [32]. There is still lack of knowledge about interaction of TcdA with specific receptors. Additionally, in contrast to TcdB, the CROPs of TcdA play a role in stabilization of toxin conformation [33,34] suggesting that receptor binding of TcdA is more complex than of TcdB.

## 4. Materials and Methods

### 4.1. Expression and Site Directed Mutagenesis of Recombinant TcdB

TcdB from *C. difficile* VPI10463 and R20291 was cloned into pHIS1522 vector as described by Olling et al. [3]. The historic reference strain *C. difficile* VPI10463 was used for cloning TcdB_VPI_ and the endemic *C. difficile* strain R20291 (NAP-1, RT027) was used for cloning of TcdB_R20_. *C. difficile* strain R20291 was obtained from the Deutsche Sammlung von Mikroorganismen und Zellkulturen (DSMZ), Braunschweig, Germany (DSM 27147; NCTC 13366). TcdB_VPI_ 1-1830 (TcdB_VPI_ ΔCROP) was generated as previously described [9]. TcdB_R20_ 1-1833 (TcdB_R20_ ΔCROP) was generated by restriction digestion of pHIS1522_TcdB_R20_ with Bpu10I and BamHI and subsequent ligation of the accordingly cleaved PCR product TcdB_R20_ 1175-1833 (bp 3522-5490). Point mutations for exchange of amino acid residue 1597 in TcdB were performed via PCR using Q5^®^ Hot Start High-Fidelity DNA Polymerase (M0493, New England BioLabs, Frankfurt a. M., Germany) and mutagenic primers TcdB_VPI_ F1597S and TcdB_R20_ S1597F (Table 1) according to the instruction manual of QuikChange II Site-Directed Mutagenesis Kit (Agilent Technologies, Santa Clara, CA, USA). TcdB fragments 1101–1836 were amplified from TcdB full-length constructs (pHIS1522_TcdB) with primers shown in Table 1 and ligated into pQE30 via BamHI and KpnI.

Expression of recombinant proteins was done in *Bacillus megaterium* expression system (MoBiTec, Germany) except TcdB fragments 1101-1836, which were expressed in *E. coli*. Therefore, the plasmids pHIS1522_TcdB_VPI_ F1597S and pHIS1522_TcdB_R20_ S1597F were transformed into *B. megaterium* WH320 protoplasts following the protocol provided by MoBiTec. Cultures of transformed *B. megaterium* or *E. coli* were harvested, lysed by sonication and centrifuged to isolate toxins from the supernatant. After sterile filtration, the His-tagged toxins were purified by gravity flow using Protino^®^ Ni-IDA Packed Columns (Macherey-Nagel, Düren, Germany). Elution buffer was exchanged with storage buffer (50 mM NaCl, 20 mM Tris-HCl, pH 8.0) using Zeba Desalting Spin Columns (Pierce™, Thermo Scientific, Schwerte, Germany). Concentration and purity of the toxins were determined by gel electrophoresis and Coomassie staining.

### 4.2. Generation of HeLa CSPG4^−/−^ Cells and Cell Culture

HeLa WT cells were purchased from American Type Culture Collection (ATCC). HeLa CSPG4^−/−^ were generated via the CRISPR/Cas9 system as described [9]. HeLa WT, HeLa CSPG4^−/−^ and HEp-2 cells were cultivated by 37 °C and 5% CO_2_ in Minimal Essential Medium (MEM) supplemented with 10% fetal bovine serum (FBS) and 100 units/mL Penicillin and 100 units/mL Streptomycin. The cultures were passaged 2–3 times per week. Cells were seeded one day before the experiments onto 96-well plates or 24-well plates for cell rounding and competition or binding assays to achieve 60–80% confluency.

### 4.3. Immunoblot and Overlay Assay

Proteins were resolved by SDS-PAGE and transferred onto nitrocellulose membrane by semi-dry blotting. Nitrocellulose was blocked with 5% skimmed milk in Tris-buffered saline (50 mM Tris-Cl, pH 7.5, 150 mM NaCl) containing 0.2% Tween-20 (TBS-T) for 30 min. The nitrocellulose was incubated in TBS-T with first antibody overnight at 4 °C. The next day the nitrocellulose was washed three times with TBS-T and incubated with the appropriate horseradish peroxidase conjugated secondary antibody in TBS-T for one hour at room temperature. Specific signals were detected by enhanced chemiluminescence using Pierce™ ECL Western Blotting Substrate SuperSignal West Femto (Thermo Scientific, Schwerte, Germany) and documented with the Kodak Digital Science™ Image Station 440CF system. Immunoblots were densitometrically evaluated using LabImage 1D.

For overlay assay, indicated proteins (FZD2~Fc and human IgG1 Fc protein, both ACROBiosystems, Newark, DE, USA, Germany, 100 ng/µL in PBS) were directly spotted onto nitrocellulose. After blocking with 5% skimmed milk powder in TBS-T for 30 min, nitrocelluloses were incubated with 2 µg of indicated TcdB solved in TBS-T. TcdB was detected by immunoblot using polyclonal rabbit TcdB antibody.

### 4.4. Affinity Purification of Specific Anti-TcdB IgG

TcdB-antiserum was raised against non-toxic glucosyltransferase-deficient mutant TcdB_VPI_ D286/288N according to the national Protection of Animals Act (Permission No. 33-42502-03A351). Molecular cloning and recombinant expression of TcdB_VPI_ D286/288N was done as described earlier [17]. For affinity purification of specific IgG TcdB_VPI_ D286/288N was solved in buffer (200 mM NaHCO_3_, 500 mM NaCl, pH 8.5) and coupled to cyanogen bromide-activated sepharose 4B (Sigma #C9142-1G). The TcdB-coupled sepharose was given into a disposable column (Pierce #29922) afterwards. TcdB-antiserum was added to the column and polyclonal TcdB antibodies were eluted with elution buffer (100 mM Glycin pH 2.5, 1 M Tris-HCl pH 9.5). Concentration and purity of the antibodies were determined by SDS-PAGE and Coomassie staining.

### 4.5. Binding Assay

Binding assay was performed to investigate the binding of the frizzled binding domain to HeLa wildtype and CSPG4^−/−^ cells. Cells were pre-incubated at 4 °C for 15 min to prevent endocytosis. Afterwards, 4 °C cold medium supplemented with the toxin fragment containing the frizzled binding domain (RBD2) of either TcdB_VPI_ (1101–1836) or TcdB_R20_ (1101–1836) was applied to the cells for 30 min at 4 °C, to allow binding to the cell surface. The cells were washed two times with cold PBS, solved in Laemmli buffer and sonicated. Immunoblotting using polyclonal rabbit TcdB antibody was performed.

### 4.6. ELISA

Binding of FZD2 to TcdB_VPI_ and TcdB_R20_ was investigated with ELISA. The toxin was solved in coating buffer (100 mM Na_2_HPO_4_, pH 8.0) to a concentration of 10 µg/ml and coated onto a 96-well plate by applying 100 µL per well at 4 °C overnight. The plate was washed once with PBS and blocked with 1% human serum albumin (BSA) in TBS-T at room temperature (RT) for 1 h. After washing with PBS three times, the plate was incubated with 200 ng/well FZD2~Fc (ACROBiosystems, Cat.: FZ2-H5251, solved in PBS) at RT for 1 h. To evaluate toxin-bound FZD2~Fc the Fc tag was detected using peroxidase conjugated anti-hIgG antibody (Rockland, USA) solved in PBS. After incubating with peroxidase substrate o-phenylenediamine dihydrochlorid (0.04% in 200 mM Na_2_HPO_4_, 100 mM citrate acid, pH 5.0) in the dark at RT for 30 min, the individual absorptions were detected at 450 nm using the TECAN infinite^®^ 200 device (Männedorf, Switzerland) and the TECAN Magellan system (Version 6.5, 2008).

### 4.7. Evaluation of Cytopathic and Cytotoxic Effects

The potencies of TcdB-variants were determined time and concentration-dependently to derive their EC50 values. Therefore, cells were incubated with cell culture medium supplemented with indicated toxins at given concentrations and time at 37 °C and 5% CO_2_. Afterwards, for cytopathic assays, completely rounded cells were counted microscopically in randomly chosen areas at 20-fold magnification. For cytotoxic assays, cells were incubated with toxin concentrations up to 10 nM for 4 h and either pyknotic cells that show blistering or/and condensed nucleus or DAPI-positive cells per total cells were counted manually in randomly selected areas at 20-fold magnification.

### 4.8. DAPI Incorporation Assay

Cells were incubated with cell culture medium supplemented with indicated toxins at given concentrations and time at 37 °C and 5% CO_2_. Subsequently, DAPI solution was added directly to the medium at a final concentration of 300 nM. DAPI-stained cells were detected by fluorescence (excitation: 350 nM, emission: 470 nM) and phase contrast microscopy of the equal area.

### 4.9. Competition Assays

For competition assays, cells were pre-incubated with given fragments of TcdB in 1000-fold molar excess for 5 min before being challenged with indicated full-length TcdBs. Cell rounding was evaluated by microscopy as described.

### 4.10. Statistics

All data analyses were performed with GraphPad Prism 5, version 5.02, (2008). Shown are mean values ± standard deviation. For significances two-tailed unpaired student’s *t*-test was performed and *p*-values were labeled accordingly: * <0.05; ** <0.01; *** <0.001. Where indicated, non-significant differences (n.s.) were also labeled.

## Figures and Tables

**Table 1:** Oligonucleotides used for cloning of constructs and site directed mutagenesis.

Primer	Base Sequence (5′→3′)
TcdB_R20_ 1102-sense	AAGGATCCGGAATTTCAGCAGGTATACCAAGTTTAG
TcdB_R20_ 1830-antisense	AATGGATCCCATTCCAAAGTTATTAATATAAAATTTCTC
TcdB_VPI_ F1597S-sense	GAATATAAAAAGTATTTTCGTTAATTCCTTACAATCTAATATTAAG
TcdB_VPI_ F1597S-antisense	CTTAATATTAGATTGTAAGGAATTAACGAAAATACTTTTTATATTC
TcdB_R20_ S1597F-sense	GAATATAAAAAGTATTTTCATAAATTTCTTACAATCTAATACTAAG
TcdB_R20_ S1597F-antisense	CTTAGTATTAGATTGTAAGAAATTTATGAAAATACTTTTTATATTC
TcdB_VPI_ 1101-1836-sense	AAGGATCCGGAATTTCAGCAGGTATACCAAGCTTAG
TcdB_VPI_ 1101-1836-antisense	AAGGTACCTTAAGACACCATCATTCCAAAGTTATTAATATAAAATTTC
TcdB_R20_ 1101-1836-sense	AAGGATCCGGAATTTCAGCAGGTATACCAAGTTTAG
TcdB_R20_ 1101-1836-antisense	AAGGTACCTTAAGATACCATCATTCCAAAGTTATTAATATAAAATTTC

## Supplementary Materials

The following are available online at https://www.mdpi.com/2072-6651/12/12/736/s1, Figure S1: Recognition of toxins and fragments of different toxinotypes by affinity purified polyclonal antibody raised against full-length TcdB from historical strain VPI10463.
