# Supplementary material for: Receptor Binding Domains of TcdB from Clostridioides difficile for Chondroitin Sulfate Proteoglycan-4 and Frizzled Proteins Are Functionally Independent and Additive
Source: Toxins (Basel). 2020 Nov 24;12(12):736. doi: 10.3390/toxins12120736 (PMC7759879; doi:10.3390/toxins12120736)
Supplement: Supplementary file 1 [file toxins-12-00736-s001.pdf]

# Supplementary Materials: Receptor Binding Domains of TcdB from *Clostridioides difficile* for Chondroitin Sulfate Proteoglycan-4 and Frizzled Proteins are Functionally Independent and Additive

**Daniel Henkel, Helma Tatge, Dennis Schöttelndreier, Liang Tao, Min Dong and Ralf Gerhard**

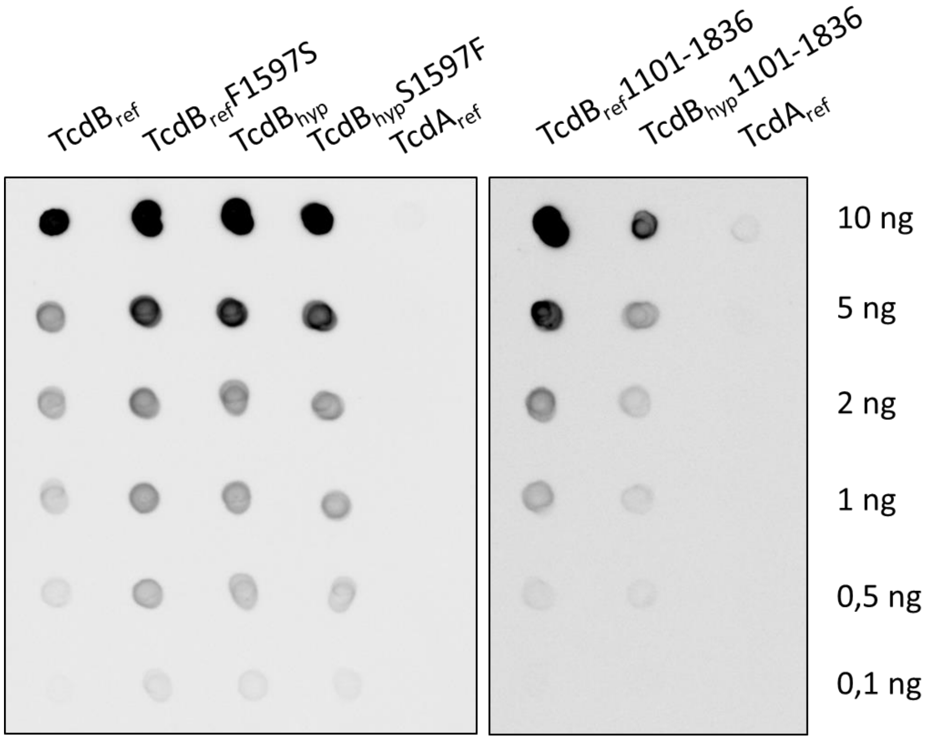


Supplementary figure 1

**Figure S1.** Recognition of toxins and fragments of different toxinotypes by affinity purified polyclonal antibody raised against full-length TcdB from historical strain VPI10463. **Method**: Indicated amount of toxins or toxin fragments were spotted onto nitrocellulose, air dried, and blocked with 5% skimmed milk powder in TBS containing 0.2% Tween-20 (TBS-T). Incubation with purified polyclonal anti-TcdB IgG (100 ng/mL in TBS-T supplemented with 1% skimmed milk powder) was performed for one hour, followed by three times washing with TBS-T. The nitrocellulose was incubated with secondary antibody (monoclonal anti-rabbit IgG, HRP conjugated) for one hour. After three times washing with TBS-T the nitrocellulose was incubated with Supersignal West Femto enhanced chemiluminescence substrate (Thermo Scientific) and signals were detected by the LAS-3000 Imaging System from Fuji. **Results:** Full-length $TcdB_{R20}$ and $TcdB_{R20}$ S1597F were recognized by anti-TcdB with a comparable specificity and sensitivity as $TcdB_{VPI}$ and $TcdB_{VPI}$ F1597S. In contrast to full-length TcdB the isolated intermediate domain aa 1101–1836 of $TcdB_{R20}$, comprising the FZD-binding domain and part of the CSPG4 binding domain, was recognized three-fold less sensitive than $TcdB_{VPI}$ 1101–1836. A weaker recognition of CROP-depleted $TcdB_{R20}$ compared with CROP-depleted $TcdB_{VPI}$ by monoclonal phage-display derived antibodies was previously observed (Chung et al., (2018) Frontiers in Microbiology, 9:2314, doi: 10.3389/fmicb.2018.02314).
